# Utilization of preventative health checkup services in China among middle-aged and older adult population: evidence from China’s 28 provinces

**DOI:** 10.3389/fpubh.2025.1500018

**Published:** 2025-02-12

**Authors:** Jingyu Shen, Weiji Fang, Yating Zhu, Chunli Ye, Yanhua Zhu, Yanling Tao

**Affiliations:** ^1^Center Operating Room, Longgang Central Hospital, Shenzhen, China; ^2^Nurse Department, Longgang Central Hospital, Shenzhen, China; ^3^Human Resources Department, Zhuhai People’s Hospital (The Affiliated Hospital of Beijing Institute of Technology, Zhuhai Clinical Medical College of Jinan University), Zhuhai, China; ^4^Medical Sciences Division, Macau University of Science and Technology, Macau, Macao SAR, China

**Keywords:** health checkup services, public health, middle-aged, older adult population, utilization

## Abstract

**Introduction:**

Research on the utilization of outpatient and inpatient medical and health services for residents and the factors influencing them is well established, however, there are fewer relevant studies analyzing the utilization of preventive health check-up services for middle-aged and older adult people in China. In this study, we hope to understand the utilization of preventive health care services and identify the factors that influence such utilization, thereby providing insights for health policy and resource allocation.

**Methods:**

The study uses data from the 2020 CHARLS survey, including 17,200 participants aged 45 and older.

**Results:**

Approximately 47.3% of middle-aged and older adult individuals had at least one health checkup. Utilization was significantly influenced by age, area of residence, education level, social insurance, health insurance, personal income, presence of chronic diseases, and life satisfaction. Older adults, urban residents, and individuals with higher income or chronic conditions were more likely to utilize health checkup services.

**Conclusion:**

There are significant urban–rural disparities in the utilization of health checkup services among middle-aged and older adult people in China. Future health policies should prioritize rural areas and disadvantaged groups to improve equity and accessibility of health services.

## 1 Introduction

### 1.1 Background

Globally, healthcare systems face mounting challenges due to aging populations and the rising prevalence of chronic diseases. The proportion of people aged 65 and older is expected to double by 2050, while non-communicable diseases (NCDs), such as cardiovascular diseases, diabetes, and cancers, have become the leading causes of death worldwide (1). This shift in disease burden is closely tied to changes in health risk factors. Traditional risks, such as environmental and occupational hazards, are increasingly being replaced by modern risks related to lifestyle and metabolism, including poor diet, physical inactivity, and obesity (2). These trends, driven by urbanization and socioeconomic development, place immense pressure on healthcare systems, demanding innovative responses from public health leaders globally.

**Figure 1:**
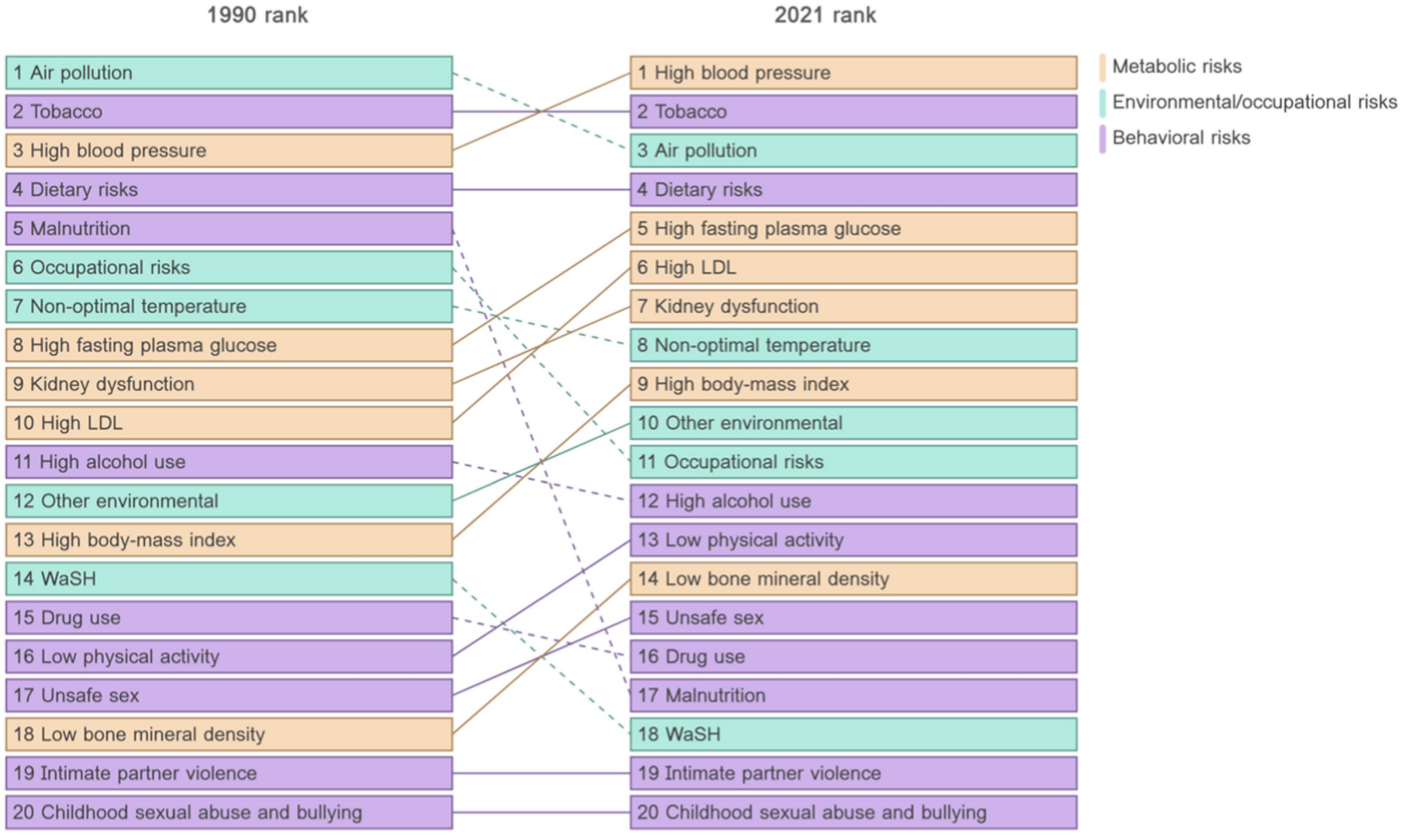
Transformation of health risks in China, 1990–2020. (Data source: The Global Burden of Disease database).

As a developing country with the largest population in the world, China is also facing the above challenges. First, along with the development of Chinese society, the risk factors affecting the health of the Chinese population have shifted from traditional factors to health behavioral factors, which is the same as the global changing trend (3–5). Our trend map based on the Global Burden of Disease database can exactly illustrate this changing trend effectively (Figure 1). Secondly, China is also facing the serious challenge of a rapidly aging population, with the proportion of older adult people in China expected to exceed 30% of its total population by 2035 (6). These dual pressures—shifting health risks and an aging population—have led to a sharp increase in chronic diseases (7, 8), straining China’s healthcare system. In 2018, total health expenditures accounted for 6.4% of the nation’s GDP, a figure projected to exceed 10% by 2030 (9). This underscores the urgent need for innovative policies to address the financial and healthcare burdens associated with these trends.

In response, China has introduced proactive policies centered on preventive care. The Basic Healthcare and Health Promotion Law, enacted in 2020, emphasizes “proactive health” management, encouraging individuals to adopt healthier lifestyles through balanced diets, regular exercise, and the avoidance of harmful habits (10). Preventative health checkup are a key component of this strategy, enabling early detection of risk factors and diseases, reducing healthcare costs, and improving outcomes (11). Through such a more proactive health management posture, China’s public health managers hope to improve people’s health literacy and self-care awareness, and at the same time provide effective reference and reference for countries facing similar challenges around the world.

However, evidence on the utilization of preventative health checkups among middle-aged and older adults in China remains limited, creating barriers for policymakers seeking to promote these services effectively. Existing studies suffer from two significant gaps. First, most research is regionally focused and lacks a comprehensive national perspective. For example, Du (12) and colleagues examined health checkup utilization among 1,200 older adults in Sichuan Province but failed to provide insights representative of China as a whole. Similarly, studies by Jiang (13) and Zhang (14) explored specific factors like supplementary health insurance and health shocks but did not address broader patterns of service utilization. Second, much of the existing research targets specific subpopulations, such as migrant workers or urban stroke patients, rather than general middle-aged and older populations. For instance, Lin (15) analyzed primary healthcare utilization among migrants, while Zhu (16) examined healthcare access among urban stroke patients. These gaps have left public health managers without adequate evidence to develop targeted strategies to improve health checkup utilization among middle-aged and older adults, potentially exacerbating health inequalities. A comprehensive, nationally representative study is therefore essential to fill these gaps and inform effective policy interventions.

### 1.2 Theoretical framework

**Figure 2:**
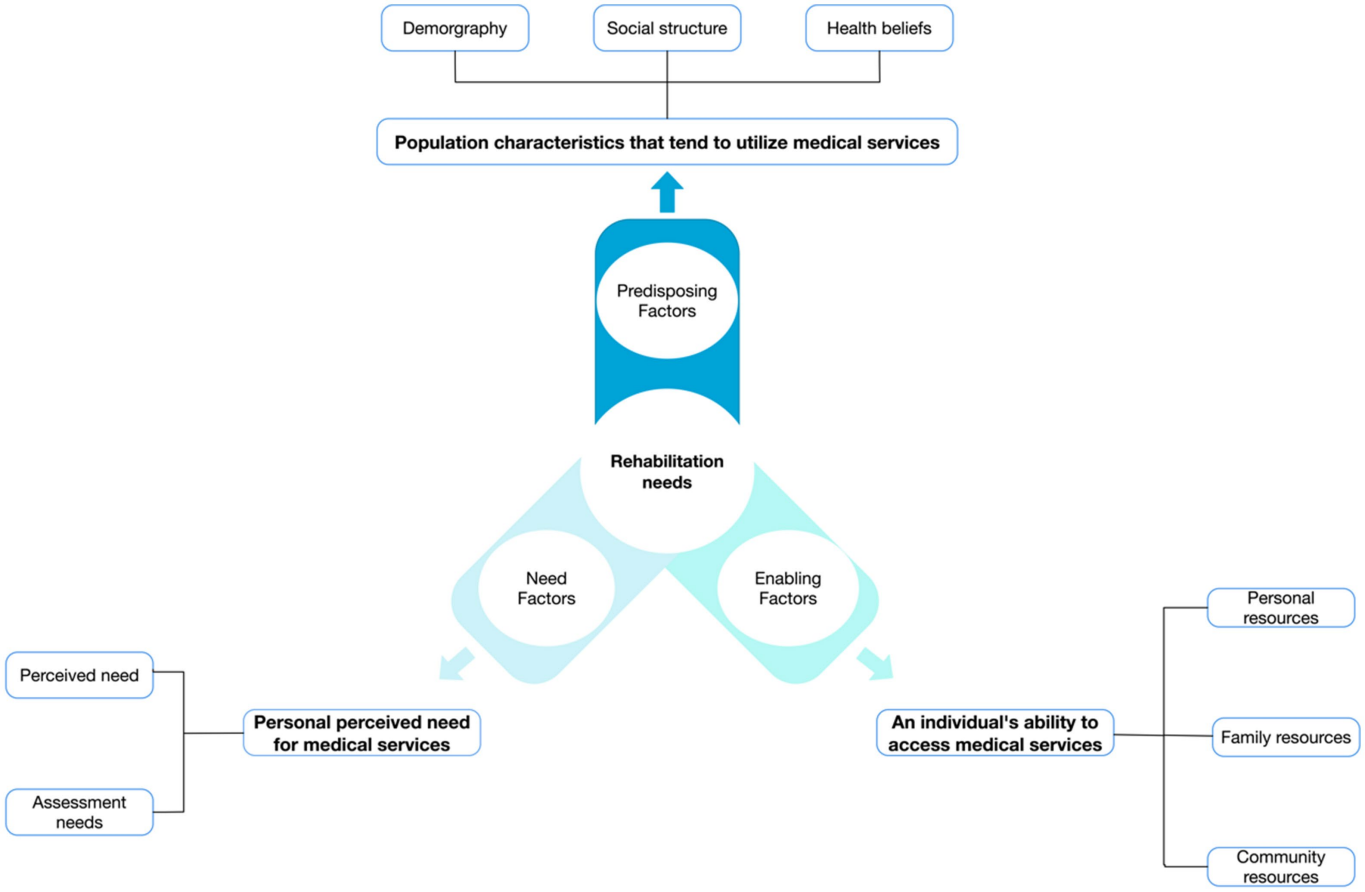
Theoretical framework of BMHSU.

The Behavioural Model of Health Services Use (BMHSU) provides a valuable framework for identifying factors that may influence health services use among middle-aged and older adults. Recognized internationally as a leading theoretical approach, the BMHSU is widely used to analyze health service behaviors. The model categorizes influencing factors into three components: predisposing factors, enabling factors, and need factors. Predisposing factors refer to characteristics such as demographics, social structure, and health beliefs that predispose individuals to seek health services, even before the onset of illness. These factors are not directly linked to service use but shape the likelihood of seeking care. Enabling factors reflect an individual’s capacity to access healthcare services and the availability of resources, including personal or family assets and community-level infrastructure. These factors indirectly affect service utilization by determining accessibility. In contrast, need factors represent the most direct influence on healthcare utilization, as they pertain to an individual’s perceived or evaluated health status and healthcare needs. Together, these three components interact to shape an individual’s decision-making process regarding health service use. The BMHSU has been successfully applied in various studies, including research on institutional aging preferences, long-term care utilization, and community health management. Its robust explanatory power makes it well-suited to exploring health service demand and utilization (17, 18). In this study, the model was used to construct a theoretical framework for identifying potential factors influencing health checkup service utilization among middle-aged and older adults. By leveraging this framework, we aimed to analyze and pinpoint the key determinants most likely to drive the use of these services (Figure 2).

## 2 Methods

### 2.1 Data source

Data used in the study were derived from China Health and Retirement Longitudinal Survey (CHARLS). CHARLS is a national longitudinal survey initiated by Peking University, which began in 2011 and continued for four more longitudinal surveys in 2013, 2015, 2018, and 2020. In this study, we used the most recently published data, which was surveyed in 2020 and publicly released in December 2023. This is a public database, all staff can be accessed through http://charls.pku.edu.cn/en, and access to application data. Detailed information on data collection is also available on this website.

### 2.2 Study population and sampling

The CHARLS collects data from a national sample of people over 45 years. In terms of sampling methodology, multi-stage stratified probability proportional to size (MPPS) sampling was used for selection, and 17,708 participants from 10,257 households were recruited from 150 counties or districts and 450 villages across 28 provinces in China in CHARLS 2011 (19). All participants were followed up every 2 years after the baseline survey. Data included individual weighted variables to ensure the survey sample was nationally representative. In the most recent data we used, surveys in some provinces were not conducted as scheduled due to epidemic constraints, so the data in our study covered about 445 sample communities in 27 provinces in China.

Data were screened according to the following exclusion criteria: (1) not age information, (2) individuals younger than 45 years of age, and (3) the presence of missing data. Finally, out of the data for all 19,395 participants, we excluded 2,195 younger than 45 years (*n* = 238) and missing data (*n* = 1957), and finally analyzed a sample of 17,200 individuals.

### 2.3 Ethical consideration

All participants provided informed consent, and the protocol was approved by the Peking University Ethics Review Board (approval number: IRB00001052-11,015).

### 2.4 Analysis variables

#### 2.4.1 Outcome variable

The utilization of health checkup services will be assessed through the question, “When did you last have a physical examination since 2018?” Those who answer “yes” to having had a physical examination within this period will be defined as active utilizers of health checkups services (Table 1).

**Table 1:** The variables included in the analysis of influencing factors of middle-aged and older adult population.

Variables	Definition/measuring tools	Variable type	Units/categories
Predisposing factors			
Gender	Middle-aged and older adult gender	Categorical	1 = Male 2 = Female
Age	Middle-aged and older adult age	Categorical	1 = 45 ~ 59 2 = 60 ~ 74 3= >74
Residence area	Residence area in the village or city/town	Categorical	1 = City center 2 = Combination zone between urban and rural areas (peri-urban) 3 = Village
Marital status	Current Marital status	Categorical	1 = Married 2 = Divorced 3 = Widowed 4 = Unmarried
Education level	Current highest level of education	Categorical	1 = Illiterate 2 = Elementary school (including Sishu/home/elementary school) 3 = Senior high school (including middle school, High school, vocational school) 4 = University or college above (including bachelor degree, master’s degree, doctoral degree)
Enabling factors			
Medical insurance	Medical insurance types	Categorical	1 = Basic Medical Insurance 2 = Commercial Medical Insurance 3 = No Medical Insurance
Social insurance			
Personal income	Person income in the past year	Categorical	1 = Low income (less than ¥32,189 or $4,500) 2 = Middle income (¥32,189 or $4,500 ~ ¥54,378 or $9,000) 3 = High income (more than ¥54,378 or $9,000)
Surviving children	Number of living children	Categorical	1 = 0 2 = 1 3 = ≥1
Needs factors			
Having chronic disease	Suffering from chronic diseases	Categorical	1 = Yes 2 = No
Number of chronic diseases	Number of existing chronic diseases	Categorical	1 = Yes 2 = No
Health status	Self-assessment of health status	Categorical	1 = Very good 2 = Good 3 = Fair 4 = Poor 5 = Very poor
Self-care ability	ADL scale measurement	Categorical	1 = Normal 2 = Disability
Life satisfaction	Self-satisfaction with current life	Categorical	1 = Completely satisfied 2 = Very satisfied 3 = Somewhat satisfied 4 = Not very satisfied 5 = Not at all satisfied

#### 2.4.2 Independent variable

With reference to the BMHSU research team identified the independent variables from the three dimensions of Predisposing factors, Enabling factors and Needs factors, the independent variables assignments are shown in Table 2 and the included independent variables are as follows:

**Table 2:** Health checkup services utilization among middle-aged and older adult people in china (*n* = 17,200).

Variables	Yes (n, %)	No (n, %)	*χ^2^*	*p*
Total				
Total	8,128 (47.30)	9,072 (52.70)		
Predisposing factors				
Gender				
Male	3,792 (47.00)	4,270 (53.00)	0.296	0.587
Female	4,802 (52.25)	4,336 (47.50)		
Age				
45 ~ 59	2,925 (37.50)	4,874 (62.50)	545.587	<0.001
60 ~ 74	4,282 (55.10)	3,492 (44.90)		
>74	921 (56.60)	706 (43.40)		
Residence area				
City Center	2,160 (53.70)	1,864 (46.30)	96.172	<0.001
Peri-urban	1,008 (48.30)	1,077 (51.70)		
Village	4,960 (44.70)	6,131 (67.60)		
Marital status				
Married	6,851 (46.70)	7,804 (53.50)	28.084	<0.001
Divorce	101 (44.10)	128 (55.90)		
Widowed	1,149 (51.50)	1,080 (49.50)		
Unmarried	27 (31.00)	60 (45.90)		
Education				
Illiterate	1,723 (47.20)	1,931 (52.80)	176.467	<0.001
Elementary school	3,398 (45.40)	4,085 (54.60)		
Middle school	1,775 (45.50)	2,103 (54.50)		
High school	963 (52.50)	872 (47.50)		
Bachelor degree or above	289 (78.10)	81 (21.90)		
Enabling factors				
Social insurance				
Yes	7,165 (48.80)	7,530 (51.20)	91.360	<0.001
Not	963 (38.40)	1,532 (61.60)		
Medical insurance				
Yes	7,828 (47.70)	7,000 (52.30)	22.858	<0.001
Not	200 (38.90)	381 (61.10)		
Personal income				
Low income	6,502 (80.00)	7,959 (55.00)	201.898	<0.001
Middle income	385 (54.20)	325 (45.80)		
High income	1,240 (61.20)	787 (38.80)		
Surviving children				
0	107 (44.80)	132 (55.20)	3.250	0.197
1	1,570 (48.60)	1,662 (51.40)		
≥2	6,451 (21.7)	7,278 (80.20)		
Needs factors				
Chronic disease				
Yes	3,441 (54.40)	2,889 (45.60)	202.826	<0.001
No	4,687 (43.10)	6,183 (56.90)		
Multimorbidity (≥2 Chronic disease)				
Yes	1,232 (56.60)	944 (43.40)	87.958	<0.001
No	6,896 (45.90)	8,128 (54.10)		
Health status				
Very good	887 (9.7)	1,160 (56.70)	23.072	<0.001
Good	1,053 (12.9)	1,133 (51.80)		
Fair	4,068 (18.7)	4,602 (53.10)		
Poor	1,532 (48.70)	1,613 (51.30)		
Very poor	588 (51.00)	564 (49.00)		
ADL				
Normal	7,499 (47.10)	8,418 (52.90)	1.742	0.187
Disability	629 (49.00)	654 (51.00)		
Life satisfaction				
Completely satisfied	380 (44.90)	467 (55.10)	45.142	<0.001
Very satisfied	2,570 (48.50)	2,724 (51.50)		
Somewhat satisfied	4,405 (48.10)	4,749 (51.90)		
Not very satisfied	589 (41.70)	823 (58.30)		
Not at all satisfied	184 (37.30)	309 (62.70)		

##### 2.4.2.1 Predisposing factors

Predisposing factors include age, gender, marital status, literacy, and type of residence.

##### 2.4.2.2 Enabling factors

Enabling factors encompass social insurance status, health insurance status, number of living children, and individual income from the previous year. The number of living children will be categorized into three groups: no children, one child, and two or more children. For personal income, we refer to the disposable income of residents in 2020 published by the National Bureau of Statistics of China. We define the lower limit of middle income as 32,189 yuan (Approximately $4,500) per capita and the upper limit as twice this amount, categorizing personal incomes into three levels: high, middle, and low (20).

##### 2.4.2.3 Needs factors

Needs factors include the presence of chronic conditions such as hypertension, dyslipidemia, diabetes or elevated blood sugar, cancer and other malignant tumors (excluding minor skin cancers), chronic lung diseases (e.g., emphysema and cor pulmonale), liver disease, heart disease, stroke, kidney disease, gastric or digestive disorders, emotional and mental health problems, memory-related disorders (e.g., dementia and cerebral atrophy), Parkinson’s disease, arthritis or rheumatism, and asthma. Individuals with any of these 16 conditions will be considered to have a chronic condition, while those with two or more will be considered to have multimorbidity.

Self-perceived health status and life satisfaction will be categorized into five levels, from very good to very poor. The ability to perform activities of daily living (ADL) will be assessed using a 12-item scale, with higher scores indicating poorer ability. Any response of “I cannot do this” for any of the 12 items will be considered an impairment in the ability to perform activities of daily living.

### 2.5 Statistical analysis

The data obtained were statistically analyzed using SPSS 29.0 software. Quantitative and compositional ratios (%) were used to describe the distribution of utilization of health checkup services among middle-aged and older adults across explanatory variables. Univariate analyses were performed using the Chi-square test to assess associations between explanatory variables and the outcome variable. Variables with statistical significance in the univariate analysis were included in the multivariate analysis. Before conducting the multivariate analysis, normality tests were performed to ensure data suitability. Before performing multivariate analysis, covariance test was performed to ensure the applicability of the data, the test mainly assesses the variance inflation factor (VIF), when the VIF is between 0.1 and 10, it can be assumed that there is no multicollinearity between the data, and logistics analysis can be performed (21). Binary logistic regression analysis was then employed to construct a hierarchical model based on theoretical frameworks, incorporating tendency factors, enabling factors, and demand factors sequentially. Odds ratio (OR) with 95% confidence (CI) were calculated to quantify associations. Differences were considered statistically significant at *p* < 0.05.

## 3 Results

### 3.1 Health checkup services utilization and univariate analysis results

**Figure 3:**
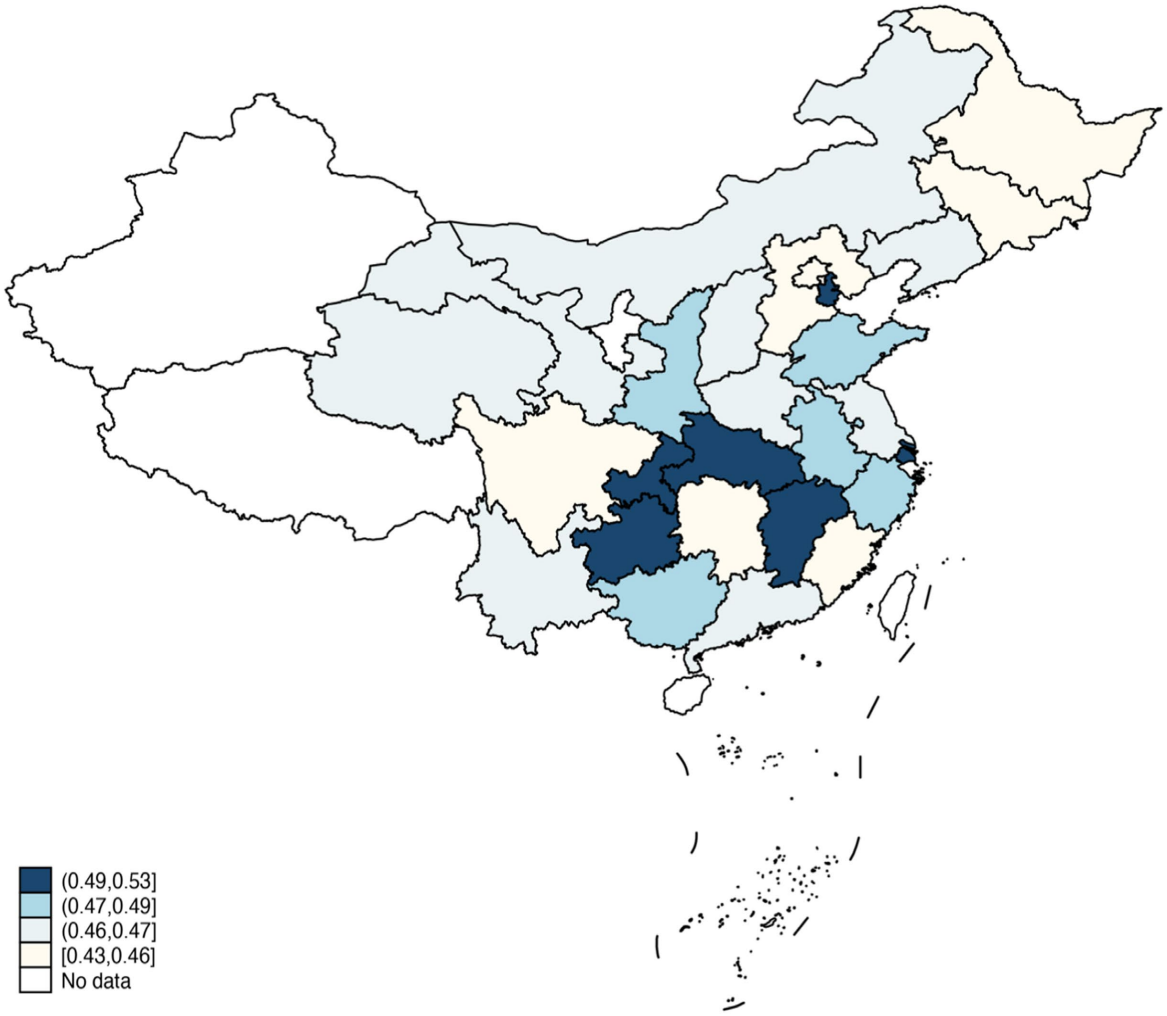
The health checkup utilization rates across Chinese provinces and cities.

In the dataset of 17,200 participants, the average age was 61.32 ± 9.33 years, with females comprising 54.46%. Since 2018, approximately 47.30% of middle-aged and older adults have undergone at least one health checkup. Figure 3 illustrates the health checkup utilization rates across Chinese provinces and cities. Further univariate analysis using chi-square tests revealed that age, residence area, education level, social insurance, health insurance, personal income, chronic diseases, multimorbidity, health status, and life satisfaction significantly influence the utilization of health checkup services. Detailed demographic information and univariate analysis results are provided in Table 2.

### 3.2 Predictors of health checkup services utilization

A covariance analysis of the predictors indicated that the variance inflation factor (VIF) for all independent variables ranged from 1.034 to 1.198, confirming no multicollinearity and suitability for logistic regression analysis. To explore the impact of various factors on health checkup service utilization among middle-aged and older adults, the study constructed three models based on the three dimensions of the BMHSU. Model 1 included four predisposing factors: age, place of residence, marital status, and education level. Model 2 expanded on this by incorporating three enabling factors: social security, health insurance, and annual personal income. Model 3 further included four need-based factors: chronic diseases, comorbidities, self-perceived health status, and life satisfaction. The classification accuracy of the models improved progressively, increasing from 52.7% in Model 1 to 60.8% in Model 3 as more variables were added. The results of Model 3 revealed that older adults (60 ~ 74, OR = 2.129, *p* < 0.001; >75, OR = 2.275, *p* < 0.001) were more likely to use health checkup services compared to middle-aged individuals. Additionally, those residing in urban areas or with higher education levels (high school or above) were more inclined to seek these services (High school, OR = 1.266, *p* < 0.001; Bachelor degree or above, OR = 3.301, *p* < 0.001). Conversely, individuals without social insurance (OR = 0.730, *p* < 0.001) were less likely to participate in health checkups. The result also showing the role of health needs. Chronic illnesses (OR = 1.449, *p* < 0.001) or Very poor self-perceived health status (OR = 1.275, *p* = 0.002) were more likely to actively seek health checkup services, emphasizing the significant influence of demand-driven factors (Table 3).

**Table 3:** Logistic regression analysis of the health checkup services utilization among middle-aged and older adults.

Variables	Model 1			Model 2			Model 3		
	OR	95%CI	*p*	OR	95%CI	*p*	OR	95%CI	*p*
Predisposing factors									
Age (Ref: 45 ~ 59)									
60 ~ 74	2.225	2.08, 2.38	<0.001	2.202	2.06, 2.36	<0.001	2.129	1.99, 2.28	<0.001
>74	2.406	2.14, 2.71	<0.001	2.370	2.10, 2.67	<0.001	2.275	2.02, 2.57	<0.001
Residence area (Ref: Village)									
City Center	1.254	1.16, 1.36	<0.001	1.172	1.08, 1.27	<0.001	1.147	1.06, 1.25	0.001
Peri-urban	1.183	1.07, 1.30	<0.001	1.169	1.06, 1.29	0.002	1.156	1.05, 1.28	0.004
Marital status (Ref: Married)									
Divorce	0.864	0.66, 1.13	0.290	0.887	0.67, 1.17	0.389	0.915	0.70, 1.21	0.528
Widowed	0.972	0.88, 1.07	0.565	1.009	0.92, 1.11	0.853	1.007	0.91, 1.11	0.893
Unmarried	0.49	0.31, 0.78	0.003	0.525	0.33, 0.84	0.007	0.512	0.32, 0.82	0.005
Education level (Ref: Illiterate)									
Elementary school	1.069	0.98, 1.16	0.115	1.05	0.97, 1.14	0.299	1.046	0.96, 1.14	0.299
Middle school	1.193	1.08, 1.32	<0.001	1.11	1.01, 1.22	0.051	1.106	1.00, 1.22	0.051
High school	1.48	1.31, 1.67	<0.001	1.26	1.11, 1.44	<0.001	1.266	1.11, 1.44	<0.001
Bachelor degree or above	4.827	3.70, 6.30	<0.001	3.34	2.53, 4.40	<0.001	3.301	2.50, 4.36	<0.001
Enabling factors									
Social insurance (Ref: Yes)									
Not				0.725	0.66, 0.79	<0.001	0.730	0.67, 0.80	<0.001
Medical insurance (Ref: Yes)									
Not				0.765	0.66, 0.89	<0.001	0.780	0.67, 0.91	0.002
Personal income (Ref: Low income)									
Middle income				1.178	1.01, 1.38	0.045	1.184	1.01, 1.39	<0.001
High income				1.599	1.43, 1.78	<0.001	1.618	1.45, 1.81	0.04
Needs factors									
Chronic disease (Ref: Not chronic disease)									
Chronic disease							1.449	1.34, 1.56	<0.001
Multimorbidity (Ref: Not Multimorbidity)									
Multimorbidity							1.108	0.99, 1.24	0.064
Health status (Ref: Very good)									
Good							1.096	0.97, 1.24	0.154
Fair							1.053	0.95, 1.17	0.327
Poor							1.088	0.96, 1.23	0.174
Very poor							1.275	1.09, 1.49	0.002
Life satisfaction (Ref: Completely satisfied)									
Very satisfied							1.12	0.96, 1.30	0.143
Somewhat satisfied							1.076	0.93, 1.25	0.330
Not very satisfied							0.871	0.73, 1.04	0.135
Not at all satisfied							0.696	0.55, 0.88	0.003

## 4 Discussion

This study utilized recent data from the China Health and Retirement Longitudinal Study (CHARLS) to explore the use of health checkup services among middle-aged and older adults in China as a form of preventive health behavior. The findings revealed that only 47.3% of participants reported undergoing health checkups, a utilization rate significantly lower than in South Korea (78.5%) and the United States (67.3–82%) (5, 22). This disparity highlights the challenges faced by a developing country like China in promoting preventive healthcare and underscores the need for improved strategies to enhance service utilization. Preventative health checkups have been shown to offer substantial health benefits and cost savings (23, 24), making it imperative for public health officials to take prompt action to increase participation rates among this demographic.

The hierarchical logistic regression analysis demonstrated that the accuracy of predicting health checkup utilization improved progressively as more predictors were added, although the magnitude of improvement varied. For instance, the accuracy increased from 52.7% in Model 1, which included only predisposing factors (e.g., age, residence, and education), to 60.6% in Model 2 with the addition of enabling factors (e.g., social insurance and income). However, the inclusion of needs factors in Model 3 led to only a slight increase in accuracy to 60.8%. These results suggest that enabling factors play a more significant role in driving health checkup utilization compared to needs factors. This is in contrast to outpatient services, where demand factors often dominate. Unlike outpatient care, which is typically sought in response to acute or chronic health conditions, preventive services like health checkups are more influenced by access to resources and susceptibility characteristics. In contrast, the proactive use of health screening services tends to depend more on favorable resources and susceptibility characteristics than on direct health needs. The results of this study also further confirm that those with health or social insurance and higher income (compared to the low-income group, the middle-income group OR = 1.178 times and the high-income group OR = 1.618) tend to be more inclined to utilize health checkup services.

The above findings suggest that enhancing accessibility and affordability of services in preventive health screening service utilization is a key lever to increase utilization. Several previous studies have analyzed the utilization of preventive care services from the perspective of private health insurance (PHI) (25), showing that PHI can probability of medical check-ups and reduce potential healthcare spending (13). This may provide us with some ideas on how public health administrators can expand the coverage of primary health insurance while at the same time supporting the coverage of supplementary insurance through more targeted measures such as direct subsidies or tax incentives (26). At the same time, public health administrators must consciously reduce the burden of screening on low-income groups and rural populations. Although China is gradually expanding free annual health screening services for people over 60 years of age, low-income or rural populations between the ages of 45 and 59 years of age should also be appropriately covered in order to avoid potential health inequities. At the same time, targeted health education campaigns may be necessary to raise awareness of the long-term benefits of regular health screening, especially for individuals without obvious or immediate health problems (27).

Another interesting finding of this study is that married middle-aged and older adults are more likely to utilize health check services than unmarried individuals. This could be attributed to the supportive effects of marriage, including financial, psychological, and social benefits, which enhance health awareness and encourage proactive healthcare behaviors (28). To leverage this insight, public health initiatives could promote family-based checkup packages and strengthen social and familial support networks to encourage greater participation.

## 5 Limitations

This study also has some limitations. Firstly, this study is a cross-sectional study, which can reflect the current situation of health service utilization among older adults, but cannot reflect the changing trend of health service utilization among middle-aged and older adults. Therefore, a more in-depth analysis using longitudinal analysis can be considered in future studies. Secondly, this study is to recognize the use of preventative health checkup services in general middle-aged and older adults, and there is no specific analysis of special groups such as mobile populations, eco-migrants, or middle-aged and older adults with disability status. Future studies can provide further insights into special populations in order to further enhance the equity and accessibility of health services. Finally, it is important to recognize that there may be some effects and interactions between different policy environments, geographic environments, and socio-ecological environments on the utilization of preventative health checkup services for middle-aged and older adults, and that future research could conduct more in-depth studies on these issues.

## 6 Conclusion

This study highlights significant gaps in the utilization of preventive health checkup services among middle-aged and older adults in China, with much room for improvement. To address these gaps, public health policymakers should prioritize resource allocation for key populations and develop targeted strategies, such as introducing supplemental insurance, providing subsidies for low-income groups, and improving access for rural residents. Enhancing the equity and accessibility of health services is essential. In parallel, public health campaigns should focus on raising awareness of the importance of preventive care, encouraging individuals to take greater responsibility for their health. Strengthening social and family support systems can also play a vital role in improving participation rates. By addressing these factors, China can make significant strides in promoting preventive health behaviors and improving overall public health outcomes.

## Data Availability

The raw data supporting the conclusions of this article will be made available by the authors, without undue reservation.
